# Phenotype and Variant Spectrum in the *LAMB3* Form of Amelogenesis Imperfecta

**DOI:** 10.1177/0022034519835205

**Published:** 2019-03-24

**Authors:** C.E.L. Smith, J.A. Poulter, S.J. Brookes, G. Murillo, S. Silva, C.J. Brown, A. Patel, H. Hussain, J. Kirkham, C.F. Inglehearn, A.J. Mighell

**Affiliations:** 1 Division of Molecular Medicine, Leeds Institute of Medical Research, University of Leeds, Leeds, UK; 2 Department of Oral Biology, School of Dentistry, St James’s University Hospital, University of Leeds, Leeds, UK; 3 School of Dentistry, Universidad de Costa Rica, Ciudad Universitaria Rodrigo Facio, San Pedro Montes De Oca, Costa Rica; 4 Cellular and Molecular Biology Centre, Universidad de Costa Rica, Ciudad Universitaria Rodrigo Facio, San Pedro Montes de Oca, Costa Rica; 5 Birmingham Dental Hospital and School of Dentistry, Edgbaston, Birmingham, UK; 6 School of Medicine, University of Leeds, Leeds, UK; 7 School of Dentistry, University of Leeds, Leeds, UK

**Keywords:** junctional epidermolysis bullosa, laminin, enamel, whole exome sequencing, X-ray computed tomography, hemidesmosomes

## Abstract

Amelogenesis imperfecta (AI) is a heterogeneous group of inherited disorders characterized by abnormal formation of dental enamel, either in isolation or as part of a syndrome. Heterozygous variants in laminin subunit beta 3 (*LAMB3*) cause AI with dominant inheritance in the absence of other cosegregating clinical features. In contrast, biallelic loss-of-function variants in *LAMB3* cause recessive junctional epidermolysis bullosa, characterized by life-threatening skin fragility. We identified 2 families segregating autosomal dominant AI with variable degrees of a distinctive hypoplastic phenotype due to pathogenic variants in *LAMB3*. Whole exome sequencing revealed a nonsense variant (c.3340G>T, p.E1114*) within the final exon in family 1, while Sanger sequencing in family 2 revealed a variant (c.3383-1G>A) in the canonical splice acceptor site of the final exon. Analysis of cDNA from family 2 revealed retention of the final intron leading to a premature termination codon. Two unerupted third molar teeth from individual IV:5 in family 2 were subject to computerized tomography and scanning electron microscopy. LAMB3 molar teeth have a multitude of cusps versus matched controls. LAMB3 enamel was well mineralized but pitted. The architecture of the initially secreted enamel was abnormal, with cervical enamel appearing much less severely affected than coronal enamel. This study further defines the variations in phenotype-genotype correlation for AI due to variants in *LAMB3*, underlines the clustering of nonsense and frameshift variants causing AI in the absence of junctional epidermolysis bullosa, and highlights the shared AI phenotype arising from variants in genes coding for hemidesmosome proteins.

## Introduction

Amelogenesis imperfecta (AI; MIM PS104500) describes a heterogeneous group of inherited conditions characterized by defective enamel formation (Smith et al. 2017). These can be inherited as autosomal dominant (AD), autosomal recessive (AR), or X-linked disorders. The genes underlying AI can be subdivided into 3 broad groups. First are genes that, when defective, cause AI alone without further phenotypic impact; examples include *MMP20* (Kim et al. 2005) and *KLK4* (Hart et al. 2004). Second is a less well-defined group including genes that, depending on the nature of the pathogenic variant, may cause AI in isolation or as part of a syndrome; genes encoding the proteins of the laminin 332 (LM332) complex typify this group. Finally, there are the genes in which pathogenic variants always cause AI within a broader clinical phenotype or defined syndrome (Crawford et al. 2007), for example, Jalili syndrome (AI and cone rod dystrophy) caused by *CNNM4* variants and enamel renal syndrome (AI, gingival overgrowth, and ectopic calcification, including nephrocalcinosis) caused by *FAM20A* variants (Parry et al. 2009; Polok et al. 2009; Jaureguiberry et al. 2012; Wang et al. 2013).

Epidermolysis bullosa (EB) is another heterogeneous group of inherited conditions characterized by skin fragility and blistering. Junctional EB (JEB; MIM 226700) forms a severe subset of EB where developmental enamel defects are always present (Wright et al. 1993). Causes for AR JEB include defects in any one of the genes encoding the heterotrimer LM332: laminin subunit beta 3 (*LAMB3*; the gene most often mutated), laminin subunit alpha 3 (*LAMA3*), and laminin subunit gamma 2 (*LAMC2*; Varki et al. 2006). Collagen type XVII alpha 1 chain (COL17A1) interacts with LM332, and *COL17A1* pathogenic variants also cause JEB (McGrath et al. 1995).

We and others showed that heterozygous variants in some of these same genes can cause hypoplastic AI without clinical skin abnormalities (McGrath et al. 1996; Murrell et al. 2007; Yuen et al. 2012; Poulter, El-Sayed, et al. 2014). *LAMB3* variants have now been reported as a cause of AD AI in 10 families (Appendix Table 1). These findings are consistent with LM332 and COL17A1 playing vital roles not only in skin structure maintenance but also in dental enamel formation.

Here we report 2 further families with AD hypoplastic AI due to variants in *LAMB3*, in the absence of mucocutaneous changes indicating EB. We also review the spectrum and distribution of published AI-causing variants and present high-resolution imaging of unerupted LAMB3 type AI teeth.

## Materials and Methods

### Patients

Individuals were recruited following informed consent in accordance with the Declaration of Helsinki and with local ethical approval (REC 13/YH/0028). Genomic DNA was obtained from saliva with Oragene DNA Sample Collection Kits (DNA Genotek) according to the manufacturer’s instructions. Unerupted third molar teeth were obtained following planned surgical extraction. Matched control teeth were obtained from the Skeletal Tissues Research Tissue Bank (School of Dentistry, University of Leeds; NRES REC 07/H1306/95+5).

### Genotyping

#### Whole Exome Sequencing and Analysis

Three micrograms of genomic DNA were prepared for whole exome sequencing with the Agilent SureSelect XT Library Prep Kit according to the manufacturer’s protocol (Agilent Technologies). Sequencing was performed on an Illumina HiSeq2500 with a 100-bp paired-end protocol. The FASTQ files were aligned to the human reference genome (GRCh37) with the Burrows-Wheeler aligner (Li and Durbin 2009). The resulting alignment was processed according to Genome Analysis Toolkit best practices (Van der Auwera et al. 2013).

Indel and single-nucleotide variants were called in the VCF format with the Haplotype Caller function of the Genome Analysis Toolkit program. With the VCFhacks package (https://github.com/gantzgraf/vcfhacks), variants present in the National Center for Biotechnology Information’s dbSNP147 or the Exome Aggregation Consortium’s database (version 0.3) with a minor allele frequency ≥0.1% were excluded. Remaining variants were annotated with the National Center for Biotechnology Information’s Variant Effect Predictor. Variants with a Combined Annotation Dependent Depletion (CADD; version 1.3) score ≥15 were prioritized, and those in genes already known to cause AI were highlighted for segregation analysis.

#### Polymerase Chain Reaction and Sanger Sequencing

Polymerase chain reaction (PCR) and Sanger sequencing of *LAMB3* exons 22 and 23 and flanking introns was carried out with HotShot Diamond MasterMix (Clent Life Sciences). Segregation analysis of variants was performed for all available family members. Primer sequences are shown in Appendix Table 2. Sanger sequencing was performed with the BigDye Terminator v3.1 kit (Life Technologies) according to the manufacturer’s instructions and resolved on an ABI3130xl sequencer (Life Technologies). Results were analyzed with SeqScape 2.5 (Life Technologies).

#### Reverse Transcriptase PCR

Blood was extracted into Tempus Blood RNA Tubes (Applied Biosystems). RNA was extracted with the Tempus Spin RNA Isolation Kit (Applied Biosystems) according to the manufacturer’s instructions. First-strand cDNA synthesis was performed with Moloney murine leukemia virus reverse transcriptase (Life Technologies) according to the manufacturer’s instructions. Primers were designed across the final 3 exons with Primer3 software (http://primer3.ut.ee/). Primers designed to amplify a product spanning an exon-exon junction within *TP53* were used as a control. PCR and Sanger sequencing were performed as before.

### Enamel Phenotyping

#### Photography

Teeth were lit from below and photographed with a Nikon D800 DSLR camera with a 105-mm Nikon lens and a Sigma ring flash.

#### Computerized X-ray Tomography

Teeth were analyzed by micro–computerized tomography (µCT) with a Skyscan 1172 (Bruker) operated at 100 kV with a source current of 100 µA and an aluminium/copper filter to reduce beam hardening. CT slices were reconstructed with Skyscan Recon software (Bruker). The CT images were calibrated with hydroxyapatite standards (0.25 and 0.75 g/cm^3^; Bruker).

Calibrated color contour maps of mineral density were generated with ImageJ (https://imagej.nih.gov/ij/?) and the interactive 3-dimensional surface plot plug-in. Videos were produced with CTVox software (Bruker).

#### Tooth Sectioning

Teeth were sectioned along the buccolingual axes with water cooling with an Accutom-5 cutter (Struers) fitted with a peripheral diamond cutting disc.

#### Scanning Electron Microscopy

After sectioning, the cut edge of each tooth was polished with 600- and 2,000-grit carborundum paper (3M), followed by a 12,000-grit nail buffer. Sections were etched by 30-s immersion in 30% phosphoric acid, followed by rinsing in excess distilled water and dried overnight under vacuum. Sections were mounted on aluminium stubs and sputter coated with gold (Agar Scientific; Elektron Technology). Microstructural analysis was undertaken with a scanning electron microscope (Hitachi S-3400N; Hitachi) operated at 20 kV with secondary electron detection.

## Results

### Patients

**Figure 1.**
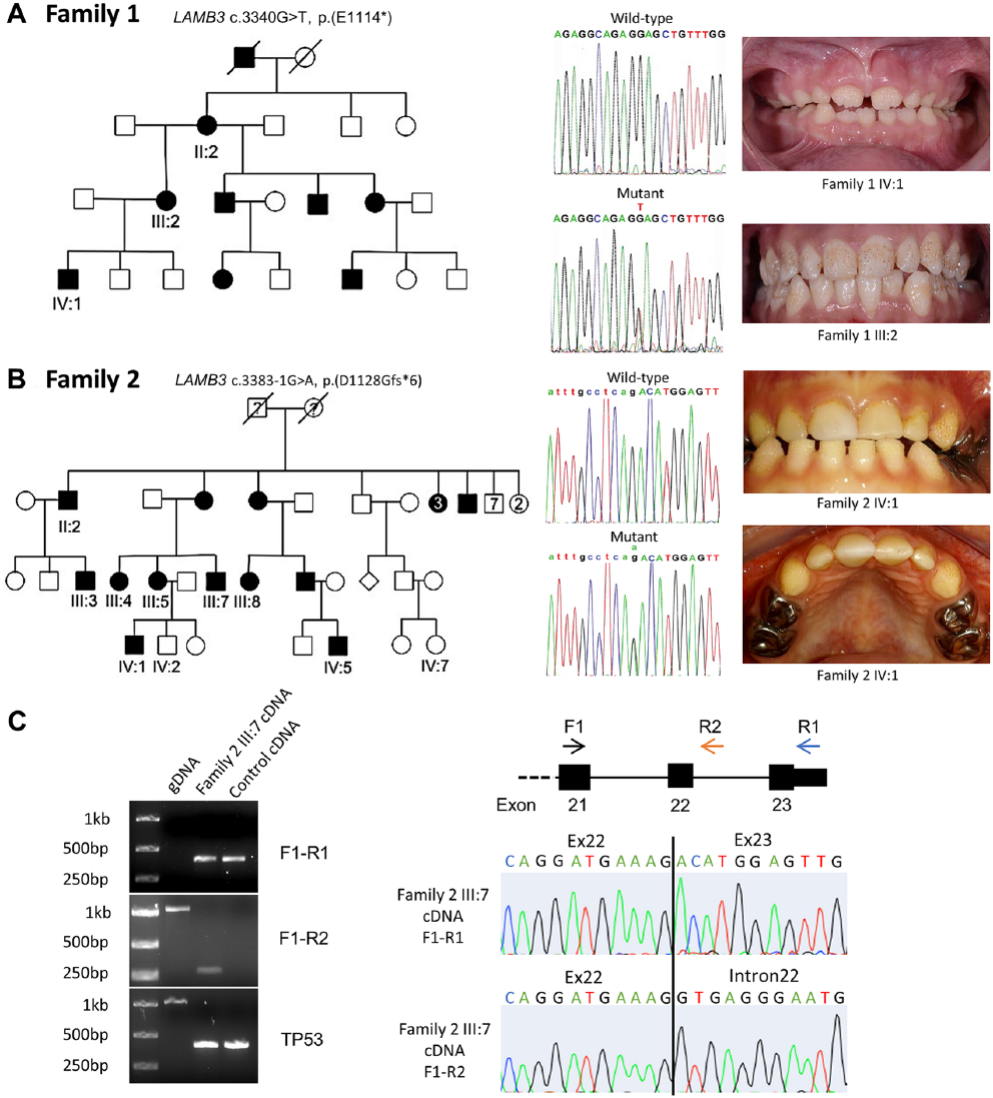
Pedigrees, sequencing traces, and splicing assay. (**A**) Pedigree for family 1, sequencing traces for laminin subunit beta 3 (*LAMB3*) c.3340G>T, p.(E1114*), and clinical images for individuals IV:1 and III:2. The generalized hypoplastic amelogenesis imperfecta (AI) in the mixed dentition of the younger individual contrasts with the more complex phenotype of the older individual, where the permanent tooth enamel is characterized by multiple pits and more subtle surface irregularities consistent with a partial reduction in enamel volume. (**B**) Pedigree for family 2, sequencing traces for *LAMB3* c.3383-1G>A, p.(D1128Gfs*6), and clinical images for individual IV:1. The unrestored primary tooth enamel is hypoplastic with multiple surface irregularities reflecting gross absence and small islands of thin enamel. All *LAMB3* variant nomenclature is based on RefSeq transcript NM_000228, RefSeq protein NP_000219.2. (**C**) Splicing assay for the *LAMB3* c.3383-1G>A variant. Amplification of cDNA from family 2 III:7 with primers located within exons 21 (F1) and 23 (F2) produced bands of 428 bp in cDNA as expected. No difference was seen between the cDNA from family 2 III:7 and the control, suggesting that only the wild-type allele was amplified. The primers were also expected to produce bands of 2,278 bp for gDNA, although it is likely that the product was too large to be amplified in the conditions used. Amplification with primers located within exon 21 (F1) and intron 22 (R2) did not produce a band for control cDNA, as expected. However, a product of 276 bp was produced from cDNA from family 2 III:7. Sequencing confirmed that the product corresponded to retention of intron 22. A gDNA control sample produced a band of 1,061 bp, as expected for this primer pair. A control polymerase chain reaction, designed to amplify *TP53* with exonic primers flanking an intron, produced a band of 410 bp for cDNA templates and 1,059 bp for gDNA templates, as expected.

A UK family and a Costa Rican family with an AD pattern of AI inheritance were recruited to the study. Both families were diagnosed with variable degrees of enamel hypoplasia, in the absence of any cosegregating health conditions (Fig. 1; Appendix Fig. 1).

### Genotyping

DNA from individual IV:1, family 1, was subjected to whole exome sequencing. Coverage statistics are available in Appendix Table 3. After filtering for rare variants in known AI genes, a heterozygous variant that creates a premature termination codon (PTC) in *LAMB3* was identified, c.3340G>T, p.(E1114*; based on NM_000228.2, NP_000219.2.) with a CADD score of 40, indicating that this is predicted to be within the top 0.01% most deleterious of all human genome variants (Appendix Table 4). The variant was confirmed by Sanger sequencing and found to segregate with disease phenotype in all available family members. This previously unreported variant is absent from dbSNP151, the Genome Aggregation Database, Exomes Aggregation Consortium (version 0.3), and Exon Variant Server. The variant alters a base within the penultimate exon (exon 22), 43 bp from the final exon junction. The truncated *LAMB3* transcript produced would be predicted to escape nonsense-mediated decay (NMD; Isken and Maquat 2007).

The distinctive phenotype observed in family 1 and in a previously reported LAMB3 AI family (Poulter, El-Sayed, et al. 2014) was noted in another AI family in the Leeds University AI genetics group study cohort, referred to hereafter as family 2. Given that the majority of AI-causing *LAMB3* variants reported to date are in the final exon of the gene (Appendix Table 1), the final coding exon (exon 23) and the flanking intron of *LAMB3* were sequenced by PCR and Sanger sequencing in this family. This revealed a previously unreported variant, c.3383-1G>A, in the canonical splice acceptor site for exon 23, which is also absent from variant databases. Sequencing of the variant in all available family members confirmed segregation with the disease phenotype.

Variants that alter the nucleotides within canonical splice sites can interfere with the accurate excision of an intron, leading to exon skipping, intron retention, or, in some cases, utilization of a cryptic splice site (Baralle and Baralle 2005). The c.3383-1G>A variant substitutes the conserved G of the AG splice acceptor site consensus sequence for the final exon of the gene. Its CADD score was 24.2, indicating that it is within the top 0.5% most deleterious of all human genome variants (Appendix Table 4). In silico analysis of the impact on splicing with Human Splicing Finder (Desmet et al. 2009) suggested that the usual splice acceptor site was disrupted by this variant (Appendix Fig. 2). To determine the effect of the variant on splicing, RNA from peripheral blood from an affected individual from family 2 (III:7) was reverse transcribed into cDNA. Primers were designed spanning the final 3 coding exons (exons 21 to 23: primers F1 and R1; Appendix Table 2) of *LAMB3*, and reverse transcriptase PCR was performed alongside control blood cDNA and a genomic DNA control. There was no difference between the control and affected cDNA, and sequencing revealed only the presence of the wild-type cDNA sequence (Fig. 1C). A primer was then designed within the final intron (intron 22: primer R2; Appendix Table 2) of *LAMB3*, and reverse transcriptase PCR was performed in combination with primer F1 in exon 21 (Fig. 1C) to test whether intron 22 was retained in the cDNA. A product was obtained from affected cDNA but not control cDNA. A much larger band of the correct size for genomic DNA was observed in the control genomic DNA. Sequencing the affected cDNA band confirmed that the sequence from the final *LAMB3* intron was present in the mRNA. Retention of this intron is predicted to result in a frameshift from amino acid 1128 leading to a PTC 6 residues later, p.(D1128Gfs*6).

Both c.3340G>T and c.3383-1G>A variants were submitted to ClinVar (SCV000537161 and SCV000537162) and the Leeds AI Leiden Open Variant Database (http://dna2.leeds.ac.uk/LOVD/; variant IDs 0000000256 and 0000000257).

### Phenotyping

Two unerupted third molar teeth (hereafter referred to as LAMB3 teeth 1 and 2) were obtained from family 2 individual IV:5 after surgical extraction. Their appearance, which was unaffected by posteruptive changes, was striking and very different to matched control teeth (Appendix Fig. 3). The occlusal surfaces of the teeth had multiple cusps separated by deep irregular pits. A deep groove encircling the tooth separated the occlusal from the cervical enamel. The cervical enamel appeared more like that of the control tooth (i.e., smooth), although it appeared that some cervical enamel had been lost during extraction.

**Figure 2.**
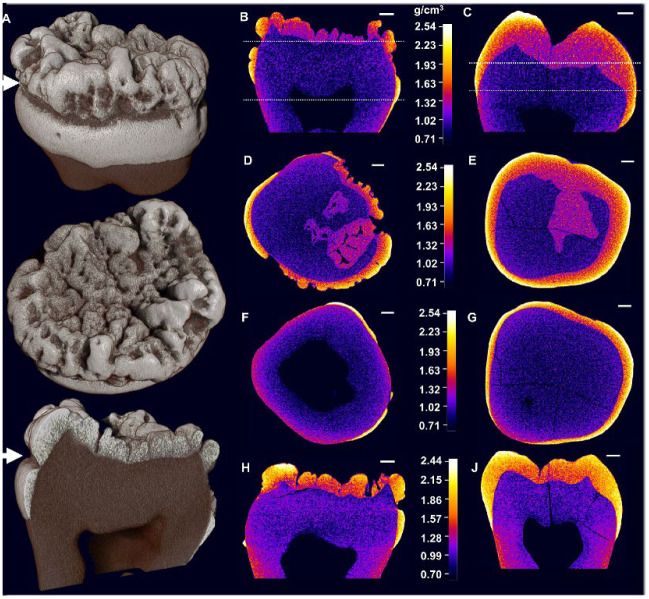
High-resolution x-ray computed tomography (CT) scan calibrated false-color plots. (**A**) Three-dimensional CT reconstruction of laminin subunit beta 3 (LAMB3) tooth 1 shows the multitude of cusps and the groove encircling the crown and separating the occlusal and cervical enamel (white arrows). (**B**, **C**, **H**, **J**) Longitudinal views through each tooth. Dotted lines on panels B and C indicate the positions of the transverse views through the coronal and cervical enamel shown in panels D–G. Transverse views through the (**D**, **E**) coronal enamel and (**F**, **G**) cervical enamel. A, B, D, F: LAMB3 tooth 1. C, E, G: control tooth 1. H: LAMB3 tooth 2. J: Control tooth 2. Note that the undulating enamel-dentine border in the affected LAMB3 teeth is not atypical; it is present in other CT sections of control teeth, as shown in Appendix Figure 6. Scale bars: 1 mm.

Three-dimensional µCT reconstructions of LAMB3 teeth illustrated the gross phenotype (Fig. 2A; Appendix Videos), while µCT sections (calibrated for mineral density) of LAMB3 teeth (Fig. 2B, D, F, H) and matched controls (Fig. 2C, E, G, J) revealed that the LAMB3 enamel was present as a hypoplastic and variable layer throughout the cusp regions. More cusps were present than in control teeth, and the enamel contained pits, some of which extended to the enamel-dentine junction (EDJ). Examination of the underlying dentine showed that the excessive number of small cusps seen in the enamel was not reflected in the dentine.

**Figure 3.**
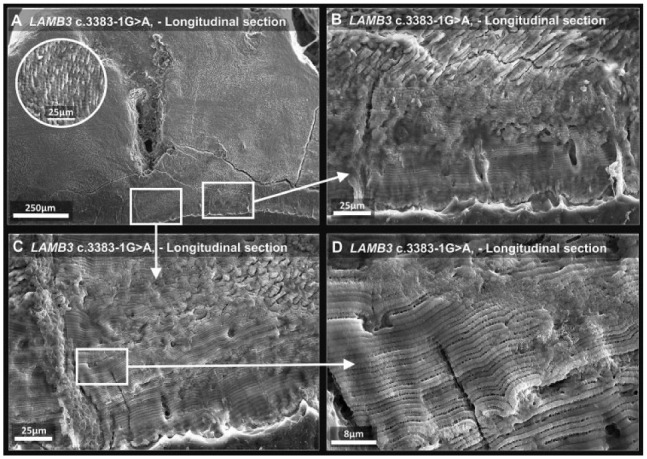
Scanning electron microscopy of laminin subunit beta 3 (LAMB3) tooth 1. (**A**) Low-power image of affected enamel shows normal prismatic enamel in the bulk tissue away from the enamel-dentine junction; inset shows a higher-magnification image of the prismatic structure present. (**B**–**D**) The white squares indicate regions scanned at increasingly higher magnification. The initially secreted enamel, nearest the enamel-dentine junction, is aprismatic and appears to be composed of lamellae. For comparison, enamel from a control tooth is shown in Appendix Figure 4.

LAMB3 tooth 2 and a matched control tooth were sectioned along the buccolingual axis. Scanning electron microscopy of control enamel showed the classic prismatic architecture (Appendix Fig. 4). Much of the bulk enamel of LAMB3 tooth 2 also exhibited normal prismatic architecture (Fig. 3A). However, near the EDJ, the initially secreted enamel was abnormal and more reminiscent of stacks of lamellae roughly 1 to 2 µm in thickness running horizontal to the EDJ (Fig. 3B, C).

## Discussion

LAMB3 is part of the LM332 complex, a component of hemidesmosomes, structures that link cells to extracellular matrices. LM332 is assembled in the rough endoplasmic reticulum, where disulphide-linked dimers of LAMB3 and LAMC2 subunits form heterotrimers with the LAMA3 subunit (Matsui et al. 1995). Only complete heterotrimers can be secreted (Matsui et al. 1995), and the C-terminal domain of LAMB3 is the part of the protein in contact with LAMA3 and LAMC2 (Rousselle and Beck 2013).

**Figure 4.**
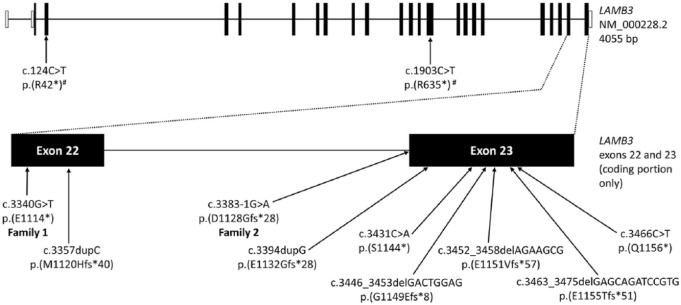
Heterozygous laminin subunit beta 3 (*LAMB3*) variants identified in individuals with amelogenesis imperfecta. Schematic diagram of the *LAMB3* transcript showing the relative positions of the disease-causing variants identified in individuals with amelogenesis imperfecta (numbering based on *LAMB3* RefSeq transcript NM_000228.2, LAMB3 RefSeq protein NP_000219.2). Note that the majority of variants identified are frameshift or nonsense changes likely to escape nonsense-mediated decay as they are positioned within the penultimate (22) exon, <50 bp from the final exon-exon junction in the mature transcript, or are within the final (23) exon or intron (22i) and have been shown to affect splicing. Two variants lie outside exons 22 and 23 (marked with #), but analysis of segregation of each variant with disease was absent or conflicting; therefore, the variants remain to be confirmed as the cause of disease.

Over 87 putative loss-of-function variants of *LAMB3* have been linked to AR LM332-deficient severe JEB (Pulkkinen et al. 1994; Kiritsi et al. 2013). The majority are variants generating PTCs, with the resulting transcripts likely to undergo NMD, with very little, if any, translated protein produced. In contrast, the *LAMB3* variants reported here are only the 10th and 11th identified as being causative of hypoplastic AD AI without clinical mucocutaneous abnormalities (Appendix Table 1, Fig. 4). With the exception of 2 variants reported by Prasad et al. (2016), all AI-causing *LAMB3* variants are close to the C-terminus and are predicted to create PTCs at positions that are unlikely to trigger NMD (Isken and Maquat 2007). Unlike the loss-of-function variants associated with recessively inherited JEB, these AI-causing variants are likely to be expressed and potentially cause isolated AI pathology through a dominant negative effect, possibly through endoplasmic reticulum stress or dysfunctional assembly and/or function of the heterotrimeric LM332 complex.

The *LAMB3* variants reported by Prasad et al. (2016) in AD AI included 2 that were also identified in patients with JEB as recurrent variants at hypermutable CpG sites (Kivirikko et al. 1996), namely c.1903C>T, p.(R635*), which accounts for 45% to 63% of all variants identified in *LAMB3* in patients with JEB, and c.124C>T, p.(R42*; Kiritsi et al. 2013). For the former, segregation of the variant with the dental phenotype was reported to be inconsistent, and for the latter, only 1 affected individual was recruited (Prasad et al. 2016). Further study is therefore required to determine whether these variants consistently give rise to a dental phenotype in JEB carriers. Given the large number of patients with JEB reported with each variant, the lack of reporting a dental phenotype in carrier relatives suggests that these variants do not routinely cause AI in isolation.

It is unclear why there are differences in clinical appearances of the anterior permanent teeth between family 1 individuals IV:1 (son) and III:2 (mother; Fig. 1, Appendix Fig. 1). The younger individual has a generalized irregular hypoplastic AI phenotype in both dentitions that is consistent with the first report (Poulter, El-Sayed, et al. 2014) and subsequent reports of AI in isolation due to *LAMB3* variants (Appendix Table 1, Fig. 4). Pitting and grooving have been reported, but a contrasting difference in clinical presentation between first-degree relatives with the same genetic variant has not been so clearly demonstrated. Clinically obvious enamel pits (of varying prominence) are features of AI arising from variants in genes encoding other hemidesmosomal proteins, including *LAMA3, ITGB6*, and *COL17A1* (McGrath et al. 1996; Yuen et al. 2012; Poulter, Brookes, et al. 2014). Accordingly, AI linked to hemidesmosome components can have a clinical presentation that is characterized by variable degrees of hypoplasia, from small irregular deposits of enamel to much greater volumes of enamel and clinically obvious pits or grooves, with intrafamily variation.

The unerupted LAMB3 molar teeth reported here provided a clear insight into enamel structure and crown morphology unaffected by posteruptive changes. The obvious differences between LAMB3 teeth and controls are the presence of multiple cusps (vs. the 4 or 5 in third molars; Nanci 2012) and the appearance of a groove separating occlusal and cervical enamel, suggesting that LAMB3 is involved in determining final cusp architecture. A recent report by Kim et al. (2016) of a 6-y-old boy with a heterozygous *LAMB3* variant, c.3452_ 3458delAGAAGCG, p.(E1151Vfs*57), showed that posterior teeth with their more complex cusp architecture were affected with hypoplastic pits and grooves, whereas anterior teeth were minimally affected. The c.3383-1G>A, p.(D1128Gfs*6) variant identified here in family 2 and the majority of other variants in *LAMB3* causing AI truncate the C-terminal domain, suggesting that this domain is essential for proper enamel cusp formation. Clinical radiographs of patients with AI carrying similarly truncating *LAMB3* variants, first published by Wang et al. (2015) and Kim et al. (2013), show unerupted third molars with multiple cusps and an encircling groove, mirroring the family 2 phenotype described here (Appendix Fig. 5).

One hypothesis explaining the formation of multiple cusps is that mutated LAMB3 affects secondary enamel knot patterning, due to loss of normal function or a gain of toxic function. Interestingly, LAMB3 is expressed strongly in the enamel knot during the cap stage of tooth development, unlike the LAMA3 and LAMC2 subunits of LM332 (Yoshiba et al. 1998a), potentially suggesting a role independent from the LM332 heterotrimer. If, as widely believed, each secondary enamel knot is associated with the development of 1 cusp (Thesleff et al. 2001), disorganization or fragmentation of secondary enamel knots could lead to the formation of additional cusps. However, the dentinal cusp patterning appears unaffected by the *LAMB3* variant. It may be that islands of secretory stage ameloblasts arising from the secondary enamel knots fail to coalesce and cannot form a contiguous enamel layer, thus generating the multiple enamel cusps observed here (Appendix Fig. 6).

The phenotypic data also suggest that LAMB3 plays a role in early amelogenesis as the initially secreted LAMB3 enamel is abnormal (Fig. 4). This is temporally separated from any role that LAMB3 might play in cusp patterning and suggests 2 distinct roles for LAMB3. Immunohistologic analysis of rodent amelogenesis showed that expression of LM332 subunits peaks during secretion and that immunostaining is strongest in the Tomes’s process (Yoshiba et al. 1998b), suggesting that it acts to tether the ameloblasts to the enamel matrix. If mutant *LAMB3* affects secretory ameloblast-matrix interactions, this may underlie the abnormal enamel architecture seen near the EDJ. Observations here suggest that once the ameloblasts have secreted around 100 µm of matrix, the subsequent enamel then exhibits typical prismatic architecture. This suggests that any effect on the structure of the enamel formed by the mutant LAMB3 is transitory.

In summary, we describe 2 novel C-terminal variants in *LAMB3* causing AD AI in the absence of skin or mucous membrane abnormalities. Phenotyping of unerupted affected third molars showed the abnormal presence of multiple cusp-like structures, whereas cervical enamel, below a groove encircling the crown, appeared unaffected. The prismatic enamel structure was also abnormal near the EDJ. These findings suggest a role for LAMB3 in the development of enamel cusp morphology and prism formation in initially secreted enamel. The data highlight a distinctive phenotype and clear phenotype-genotype correlation for AI due to variants in *LAMB3* and emphasize the clustering of AD AI pathogenic variants within the penultimate and final exons.

## Author Contributions

C.E.L. Smith, J.A. Poulter, S.J. Brookes, A.J. Mighell, contributed to conception, design, data acquisition, analysis, and interpretation, drafted and critically revised the manuscript; G. Murillo, contributed to conception and data acquisition, drafted and critically revised the manuscript; S. Silva, C.J. Brown, A. Patel, H. Hussain, contributed to design and data acquisition, drafted and critically revised the manuscript; J. Kirkham, contributed to design and data interpretation, drafted and critically revised the manuscript; C.F. Inglehearn, contributed to conception, design, data interpretation, drafted and critically revised the manuscript. All authors gave final approval and agree to be accountable for all aspects of the work.

## Supplemental Material

DS_10.1177_0022034519835205 – Supplemental material for Phenotype and Variant Spectrum in the *LAMB3* Form of Amelogenesis Imperfecta Supplemental material, DS_10.1177_0022034519835205 for Phenotype and Variant Spectrum in the *LAMB3* Form of Amelogenesis Imperfecta by C.E.L. Smith, J.A. Poulter, S.J. Brookes, G. Murillo, S. Silva, C.J. Brown, A. Patel, H. Hussain, J. Kirkham, C.F. Inglehearn and A.J. Mighell in Journal of Dental Research
