# Supplementary material for: Phenotype and Variant Spectrum in the LAMB3 Form of Amelogenesis Imperfecta
Source: J Dent Res. 2019 Mar 24;98(6):698–704. doi: 10.1177/0022034519835205 (PMC6535922; doi:10.1177/0022034519835205)
Supplement: DS_10.1177_0022034519835205 – Supplemental material for Phenotype and Variant Spectrum in the LAMB3 Form of Amelogenesis Imperfecta [file DS_10.1177_0022034519835205.pdf]

**Phenotype and variant spectrum in the *LAMB3* form of amelogenesis imperfecta**

C.E.L. Smith, J.A. Poulter, S.J. Brookes, G. Murillo, S. Silva, C.J. Brown, A. Patel, H. Hussain, J. Kirkham, C.F. Inglehearn and A.J. Mighell.

| Origin | Position (hg19/GRCh37) Chr1: | Exon/ intron (i) | Variant | Predicted amino acid change | Family |
|---|---|---|---|---|---|
| N/S: patients recruited from France, Germany and Morocco for study | g.209823368G>A | 3 | c.124C>T[#] | p.(R42*) | Prasad et al. (2016) |
| N/S: patients recruited from France, Germany and Morocco for study | g.209799066G>A | 14 | c.1903C>T[#] | p.(R635*) | Prasad et al. (2016) |
| UK | g.209789858C>A | 22 | c.3340G>T[‡] | p.(E1114*) | This study: Family 1 |
| N/S: South Korean study | g.209789841dupG | 22 | c.3357dupC[†] | p.(M1120Hfs*40) | Lee et al. (2014) |
| Costa Rica | g.209788753C>T | 22i | c.3383-1G>A | p.(D1128Gfs*6) | This study: Family 2 |
| Irish | g.209788741dupC | 23 | c.3394dupG | p.(E1132Gfs*28) | Poulter et al. (2014) |
| N/S: patients recruited from Turkey / Iran | g.209788704G>T | 23 | c.3431C>A | p.(S1144*) | Kim et al. (2013); Kim et al. (2018) |
| N/S: patients recruited from Turkey / Iran | g.209788682_209788689 delCTCCAGTC | 23 | c.3446_3453delGA CTGGAG | p.(G1149Efs*8) | Kim et al. (2013) |
| N/S: South Korean study | g.209788660_209788672 delCACGGATCTGCTC | 23 | c.3463_3475delGA GCAGATCCGTG | p.(E1155Tfs*51) | Lee et al. (2014) |
| Chinese | g.209788669G>A | 23 | c.3466C>T | p.(Q1156*) | Wang et al. (2015) |
| N/S: South Korean study | g.209788677_209788683 delCGCTTCT | 23 | c.3452_3458delAG AAGCG | p.(E1151Vfs*57) | Kim et al. (2016) |

**Appendix Table 1: Variants identified in *LAMB3* in individuals with hypoplastic AI.**
Based on *LAMB3* Refseq transcript NM_000228.2, LAMB3 Refseq protein NP_000219.2.
[#] Segregation data absent or conflicting for the reports of these variants.
[†] Variant leads to generation of a premature stop codon in exon 23.
[‡] Variant lies within 43 bases of the final exon-intron junction, therefore nonsense transcript may escape nonsense mediated decay.

| Region | Forward primer (5'-3') | Reverse primer (5'-3') | Size/bp (Template) |
|---|---|---|---|
| *LAMB3* ex22 | GTGCTTTGGCCACAGGTCT | ATCTCACTCCCATAGCACGG | 340 (gDNA) |
| *LAMB3* ex23 | GTTGGGAGTCTTGGGGAGTC | AAGCATTCCAACCCAATCTG | 278 (gDNA) |
| *LAMB3* F1-R1 | AGGCATTGAGTGCCCAAG | AGGCTGTACTTTAGGCTGCA | 2278 (gDNA); 428 (cDNA) |
| *LAMB3* F1-R2 | AGGCATTGAGTGCCCAAG | GCACGGCTAGCTCCAATAA | 1061(gDNA); no product (cDNA) |
| *TP53* | AGTACTCCCCTGCCCTCAACA | CTGGAGTCTTCCAGTGTGA | 1059 (gDNA); 410 (cDNA) |

**Appendix Table 2: Primer sequences used in this study.** See Figure 1 for details of the positions of primers F1, R1 and R2.

| Family | Sample | Mean Coverage | % Coverage | | | | | | Total reads |
|---|---|---|---|---|---|---|---|---|---|
| | | | >4 | >9 | >14 | >19 | >24 | >29 | |
| 1 | IV:1 | 70.19 | 99.0 | 98.1 | 96.5 | 94.0 | 90.5 | 86.1 | 3534596745 |

**Appendix Table 3: Alignment statistics for whole exome sequencing.**
Alignment statistics were generated using the regions targetted by the Agilent SureSelectXT Human All Exon V5 capture reagent as the interval.

| Position (hg19/GRCh37) Chr1: | Variant | CADD v1.3 Scaled C score |
|---|---|---|
| g.209789858C>A | c.3340G>T‡, p.(E1114*) | 40 |
| g.209788753C>T | c.3383-1G>A, p.(D1128Gfs*6) | 24.2 |

**Appendix Table 4: Combined Annotation Dependent Depletion scaled C score for the *LAMB3* variants identified in this study.**
‡ Variant lies within 43 bases of the final exon-intron junction, therefore nonsense transcript may escape nonsense mediated decay.

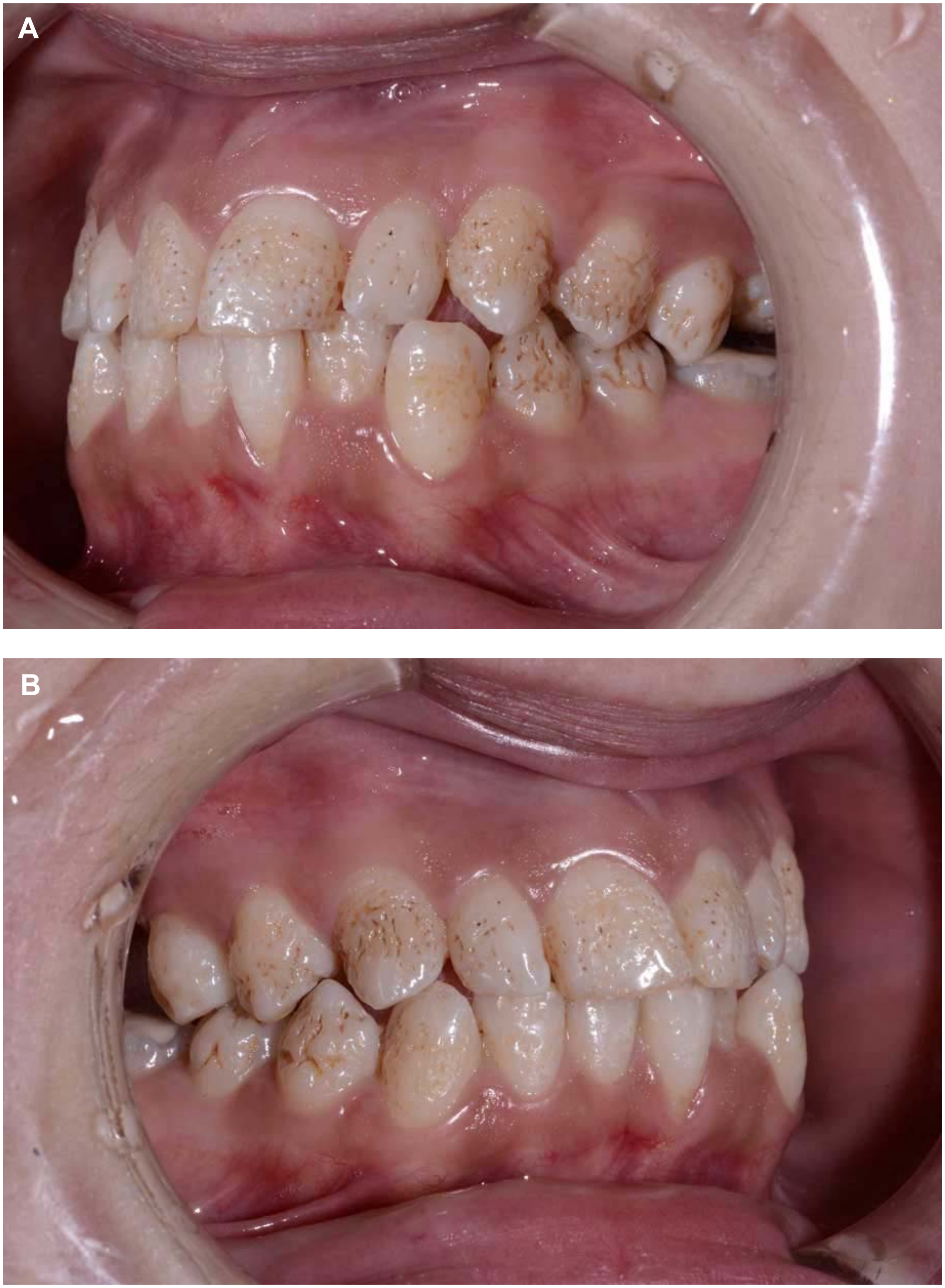
A
B

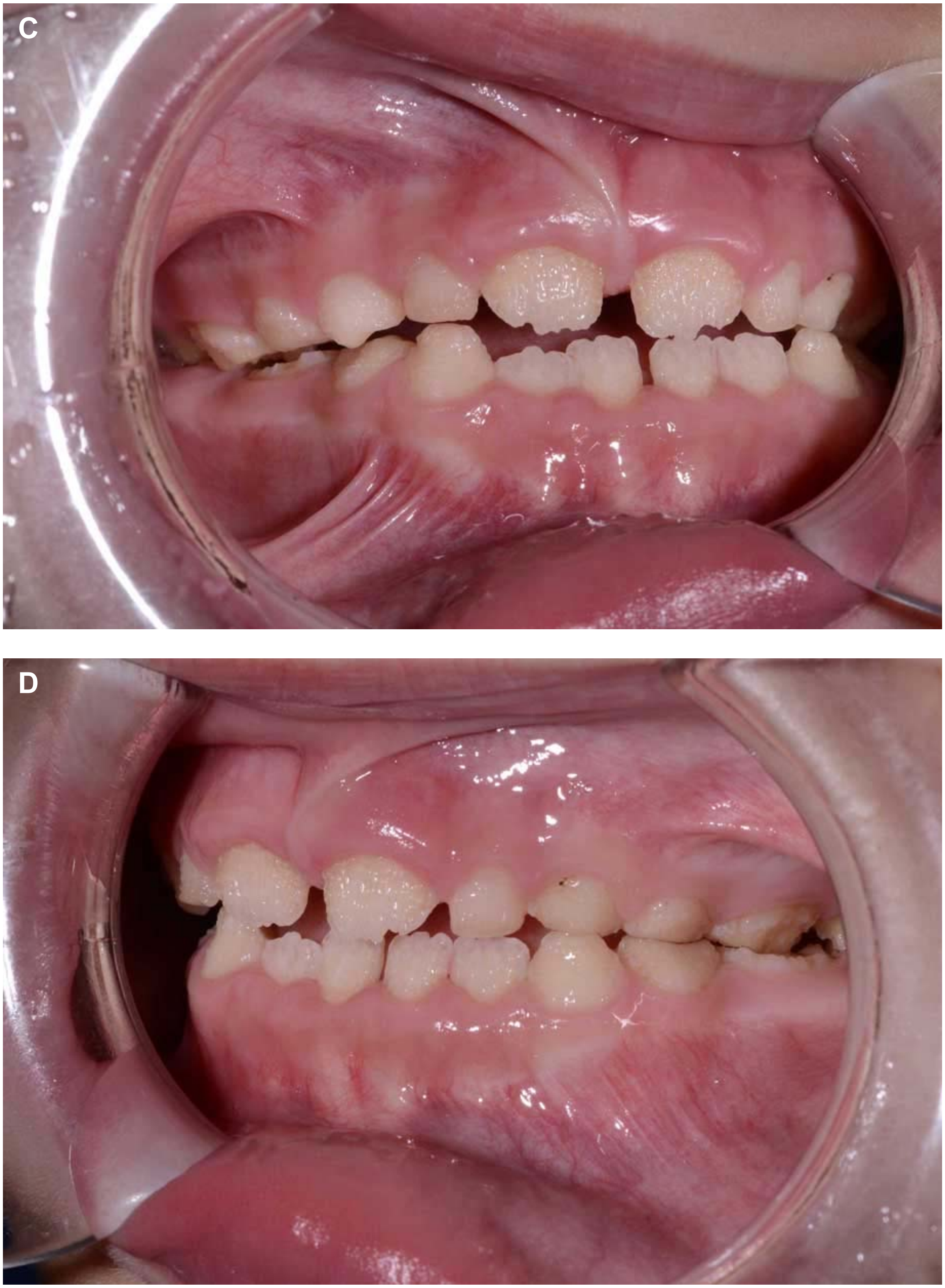
C
D

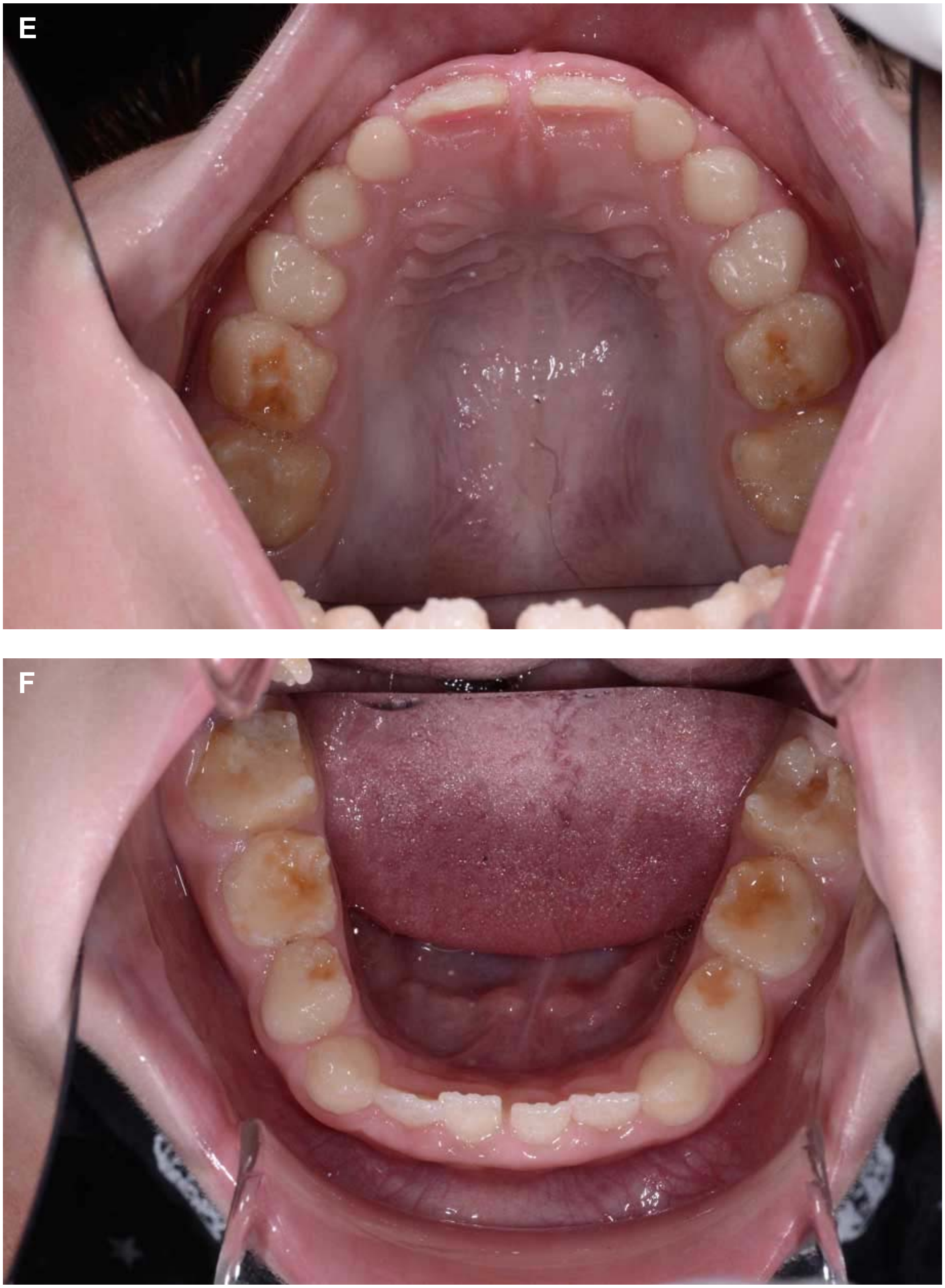


**Appendix Figure 1: Additional clinical images of family 1 III:2 (A and B) and IV:1 (C-F).**

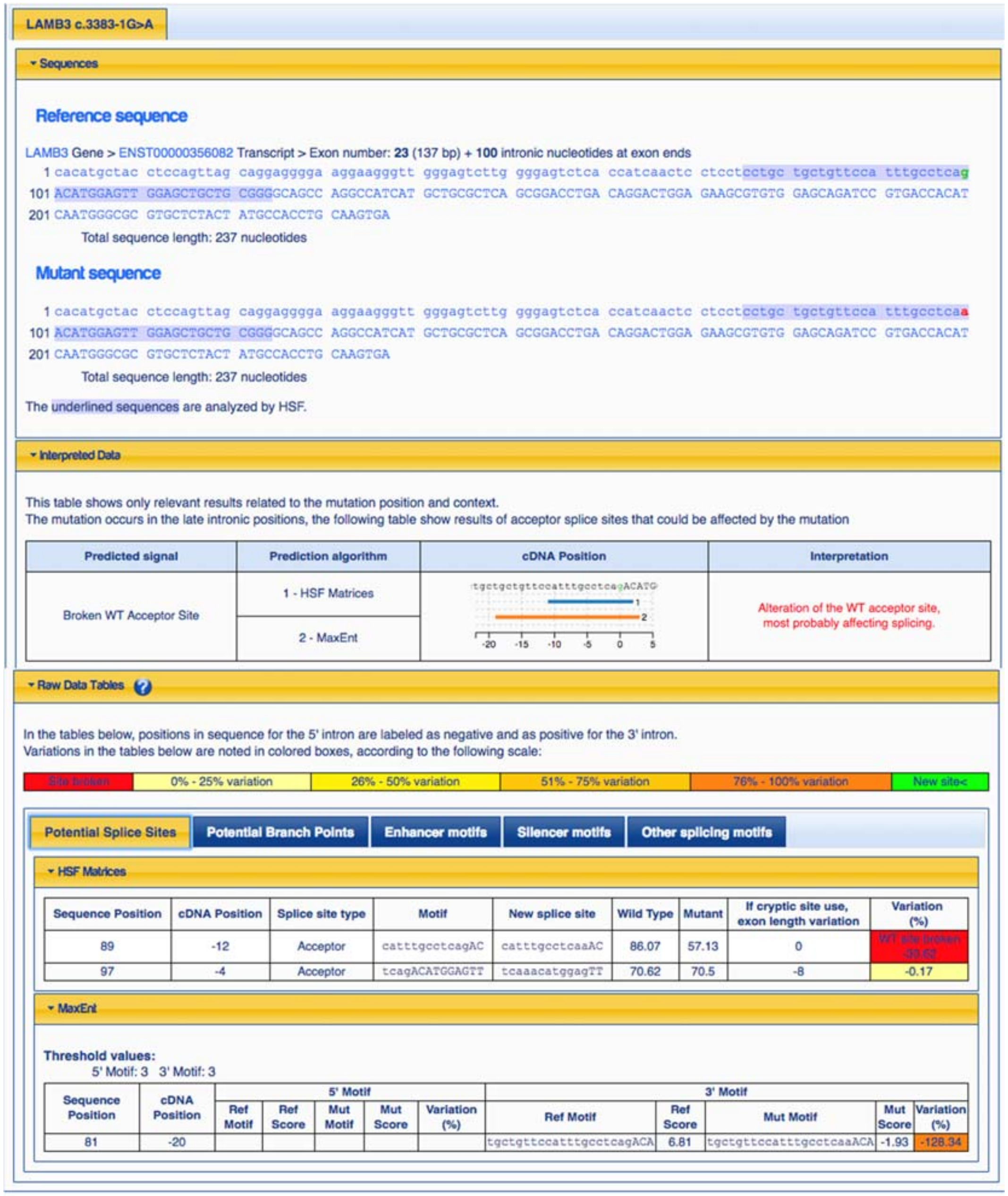


**Appendix Figure 2: Human Splicing Finder (HSF) *in silico* prediction for the *LAMB3* splice variant c.3383-1G>A identified in family 2.**

HSF predicts alteration of the WT splice site resulting in altered splicing.

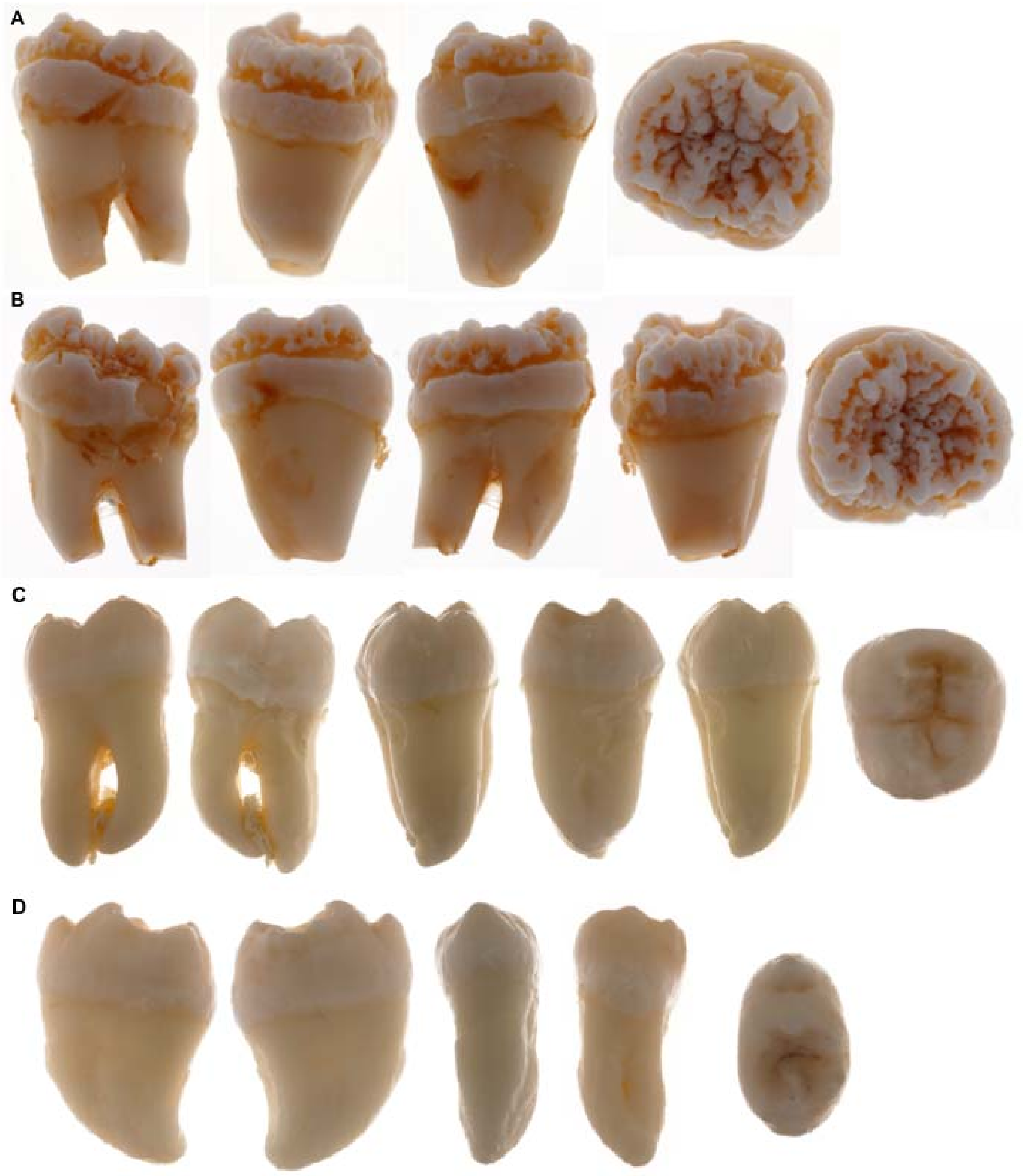


**Appendix Figure 3: Images of the two unerupted LAMB3 third molar teeth and two unerupted matched control teeth.**

A and B: Images of the LAMB3 third molar teeth surgically extracted from family 2, individual IV:5. Note the abnormal coronal enamel with multiple small, poorly formed cusps, appearing as small islands of enamel with deep, irregular pits in between. Also note the cervical ring of enamel of more normal appearance separated by a groove from the abnormal coronal enamel.

C and D: Images of matched third molar control teeth surgically extracted from unrelated individuals. Note the smooth appearance of the enamel and the defined cusp architecture.

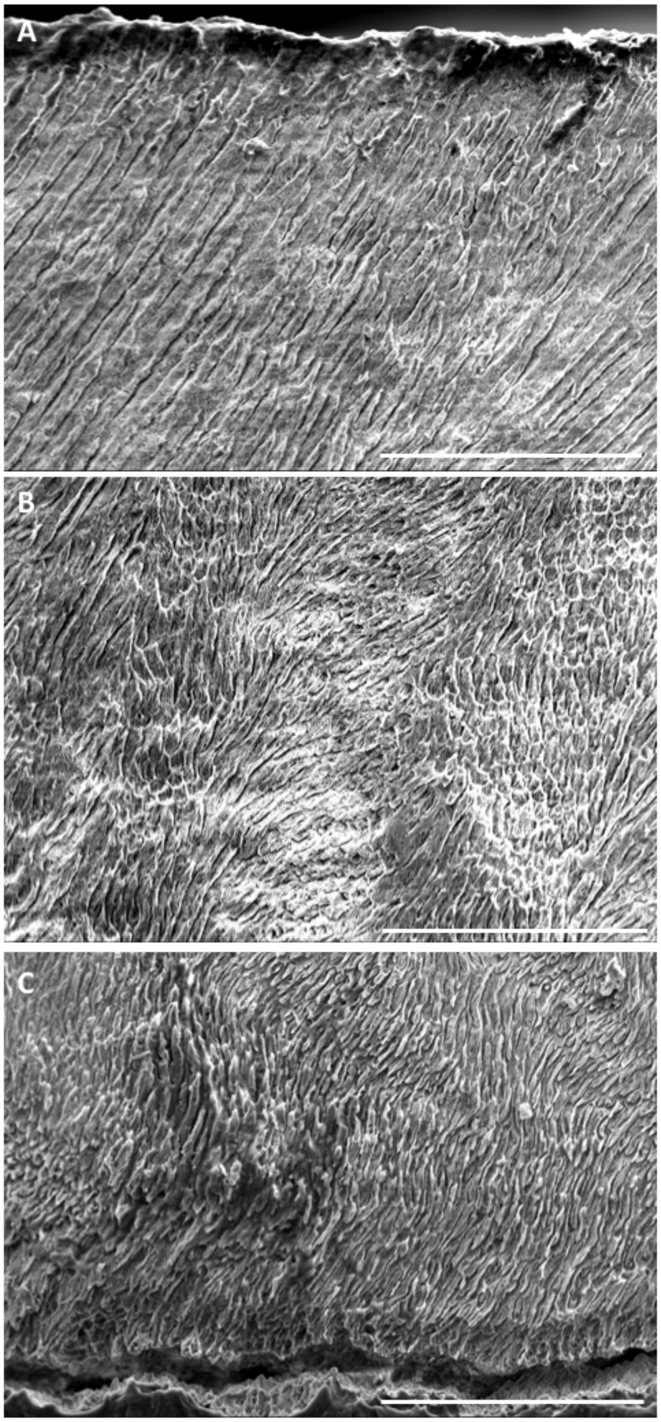


**Appendix Figure 4: SEM of control tooth 1.**

A-C: Views of the entire enamel layer, from the surface (A) to the EDJ (C). Scale bars are 100 μm.

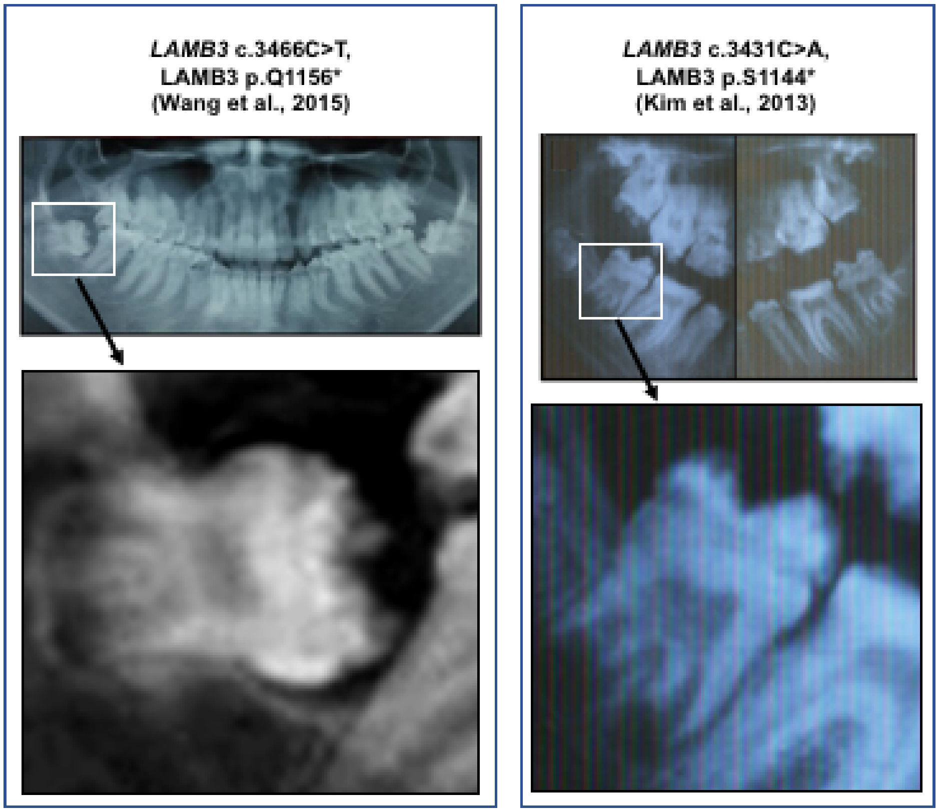


**Appendix Figure 5: Additional published reports of the enamel phenotype of individuals carrying *LAMB3* variants.**

Note the apparent presence of multiple cusps and the characteristic groove encircling the crown and separating the occlusal and cervical enamel. These features are not present on the erupted molars presumably due to post eruptive wear.

A: Images reused with permission from Wang et al. (2015).

B: Images reused with permission from Kim et al. (2013).

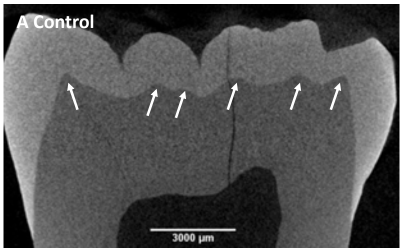


Multiple cusps at EDJ; all correspond to an enamel cusp

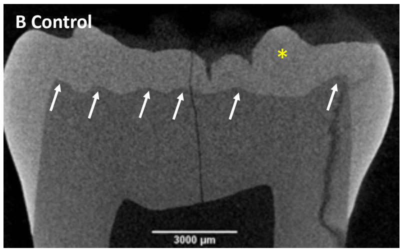


Multiple cusps at EDJ; not all correspond to an enamel cusp

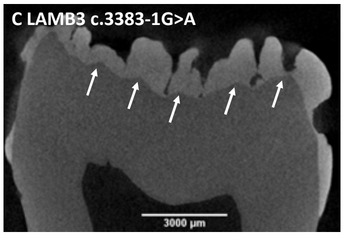


Multiple cusps at EDJ; all correspond to an enamel cusp

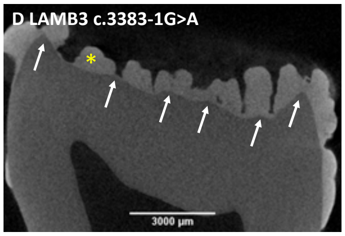


Multiple cusps at EDJ; not all correspond to an enamel cusp

**Appendix Figure 6: Additional CT images to show the presence or absence of dentine cusps below each enamel cusp.**

**A and B show different CT sections through control tooth 2. The dentine cusps at the EDJ (arrows) appear to represent the original position of the enamel knots giving rise to the various topographical features comprising the dentine surface and final crown morphology (Jussila and Thesleff 2012). It is assumed that enamel secretion also initiated at these sites and, following coalescence of islands of active secretory ameloblasts, the complete enamel thickness was deposited to generate the final topographical features of the enamel that define the final cusp morphology of the tooth. The asterisk marks an enamel cusp that is not obviously associated with an underlying dentine cusp in this CT section.**

**C and D show different CT sections through affected LAMB3 tooth 2. The EDJ is similar to the control, in that it exhibits dentine cusps (arrows) that presumably correspond to the sites of the enamel knots. However, it appears that the separate islands of active secretory ameloblasts associated with these dentine cusps failed to coalesce and continued to secrete enamel independently, leading to the formation of multiple cusps that characterise this phenotype. The asterisk marks an enamel cusp that is not obviously associated with an underlying dentine cusp in this CT section.**

**Appendix Videos:**

LAMB3 tooth 1

Control tooth 1

## References


Jussila M, Thesleff I. 2012. Signaling networks regulating tooth organogenesis and regeneration, and the specification of dental mesenchymal and epithelial cell lineages. Cold Spring Harbor perspectives in biology. 4(4):a008425.

Kim JW, Seymen F, Lee KE, Ko J, Yildirim M, Tuna EB, Gencay K, Shin TJ, Kyun HK, Simmer JP et al. 2013. Lamb3 mutations causing autosomal-dominant amelogenesis imperfecta. Journal of dental research. 92(10):899-904.

Kim YJ, Seymen F, Kang J, Koruyucu M, Tuloglu N, Bayrak S, Tuna EB, Lee ZH, Shin TJ, Hyun HK et al. 2018. Candidate gene sequencing reveals mutations causing hypoplastic amelogenesis imperfecta. Clinical oral investigations.

Kim YJ, Shin TJ, Hyun HK, Lee SH, Lee ZH, Kim JW. 2016. A novel de novo mutation in lamb3 causes localized hypoplastic enamel in the molar region. European journal of oral sciences. 124(4):403-405.

Lee KE, Ko J, Tran Le CG, Shin TJ, Hyun HK, Lee SH, Kim JW. 2014. Novel lamb3 mutations cause non-syndromic amelogenesis imperfecta with variable expressivity. Clinical genetics.

Poulter JA, El-Sayed W, Shore RC, Kirkham J, Inglehearn CF, Mighell AJ. 2014. Whole-exome sequencing, without prior linkage, identifies a mutation in lamb3 as a cause of dominant hypoplastic amelogenesis imperfecta. European journal of human genetics : EJHG. 22(1):132-135.

Prasad MK, Geoffroy V, Vicaire S, Jost B, Dumas M, Le Gras S, Switala M, Gasse B, Laugel-Haushalter V, Paschaki M et al. 2016. A targeted next-generation sequencing assay for the molecular diagnosis of genetic disorders with orodental involvement. Journal of medical genetics. 53(2):98-110.

Wang X, Zhao Y, Yang Y, Qin M. 2015. Novel enam and lamb3 mutations in chinese families with hypoplastic amelogenesis imperfecta. PloS one. 10(3):e0116514.
